# Assessment of pharmacological strategies for management of major depressive disorder and their costs after an inadequate response to first-line antidepressant treatment in primary care

**DOI:** 10.1186/1744-859X-11-22

**Published:** 2012-08-03

**Authors:** Antoni Sicras-Mainar, Jorge Maurino, Luis Cordero, Milagrosa Blanca-Tamayo, Ruth Navarro-Artieda

**Affiliations:** 1 Dirección de Planificación, Badalona Serveis Assistencials SA, Badalona, Spain; 2 AstraZeneca Medical Department, Madrid, Spain; 3 Department of Psychiatry, Badalona Serveis Assistencials SA, Badalona, Spain; 4 Department of Medical Information, Hospital Germans Trias i Pujol, Badalona, Spain

**Keywords:** Major depressive disorder, Remission, Primary care, First-line antidepressant treatment, Costs

## Abstract

**Background:**

The aim of the study was to determine the most common treatment strategies and their costs for patients with an inadequate response to first-line antidepressant treatment (AD) in primary care.

**Method:**

A retrospective cohort study of medical records from six primary care centers was conducted. Adults with a major depressive disorder diagnosis, at least 8 weeks of AD treatment after the first prescription, and patient monitoring for 12 months were analyzed. Healthcare (direct cost) and non-healthcare costs (indirect costs; work productivity losses) were described.

**Results:**

A total of 2,260 patients were studied. Forty-three percent of patients (N = 965) presented an inadequate response to treatment. Summarizing the different treatment approaches: 43.2% were switched to another AD, 15.5% were given an additional AD, AD dose was increased in 14.6%, and 26.7% remained with the same antidepressant agent. Healthcare/annual costs were 451.2 Euros for patients in remission vs. 826.1 Euros in those with inadequate response, and productivity losses were 991.4 versus 1,842.0 Euros, respectively (p < 0.001).

**Conclusion:**

Antidepressant switch was the most common therapeutic approach performed by general practitioners in naturalistic practice. A delay in treatment change when no remission occurs and a significant heterogeneity in management of these patients were also found.

## Background

Major depressive disorder (MDD) is one of the most common mental diseases in the general population, with an estimated annual prevalence of 5.7% [1]. MDD is a disabling disease that impairs health-related quality of life and causes an increase in healthcare resource utilization [2-6]. Some studies show that one third of costs are derived from healthcare and the highest are indirect costs associated to loss of work productivity [3,5,6].

It is usually recommended that patients with MDD are initially treated in primary care (PC) and approximately 80% of them are managed in PC only [7-10]. There is limited information on the referral rates to mental health care and referral criteria. In a study conducted in Spain, 23% of general practitioners (GPs) referred patients with major depression to the psychiatrist [11]. In a survey conducted by Villava and Caballero (2006) among more than 1,000 GPs in Spain, the mean referral rate was 24%, being higher in physicians who reported poorer training and greater demand for care [12]. As regards referral criteria, the main reasons reported were severity of the episode (87%), lack of response to treatment (41%), and express request by the patient (37%).

Regardless of which antidepressant (AD) is chosen, the final aim of therapy for MDD is to achieve a total remission of symptoms. Lack of remission has been associated with a higher risk of recurrences, more chronic depressive episodes, a shorter duration between episodes, and a worse functioning [8,13-16]. Only few studies have directly measured the financial impact of MDD, showing that costs are significantly lower when patients achieved remission of symptoms [3,5].

The introduction of newer-generation antidepressants has improved our ability to treat depression, although only 35-40% of patients will experience a remission of symptoms during an initial 8-week trial [17-19]. Four different pharmacologic approaches are available for treating patients who have experienced inadequate response to a first-line antidepressant: increasing the dose of the antidepressant, switching to a different antidepressant, combining the initial antidepressant with a second one, and augmenting the treatment with a non-antidepressant agent [20]. Definition of inadequate response is still controversial, but most experts define it as failure to achieve symptomatic remission [13,21,22].

The question of how to proceed with the next step in MDD treatment after an initially unsuccessful trial is crucial due to the diversity of therapeutic approaches available. The aim of the study was to determine the most common treatment strategies and their costs for patients with an inadequate response to first-line antidepressant in primary care.

## Methods

### Study design and data extraction

We carried out an observational, retrospective cohort study of medical records held by the health care provider, Badalona Serveis Assistencials (BSA). The study population consisted of patients from six primary health care centres managed by BSA. They cover a population of 120,000 inhabitants, mostly urban, middle-low socioeconomic status, and with a predominance of industrial workers.

Personal identification of patients was removed from the start, subsequently maintaining complete dissociation between patient identification and their clinical data, as requested by Spanish legislation protecting confidentiality of patient health data. Then, written consent was not required for this type of study. The study protocol was approved by the investigational review board of the Hospital Germans Trias i Pujol (Badalona, Spain; NCT01446692).

### Patients

All patients who met the following characteristics were included in the study: (a) aged over 18; (b) a diagnosis of major depressive disorder according to the International Classification of Primary Care (ICPC-2, code P76), either as a single (incidence) or recurrent episode (prevalence, new episode) [23]; (c) antidepressant treatment started between January 1st, 2008 and December 31st, 2009; (d) prescription meeting the criteria for a minimum adequate treatment (at least 8 weeks of AD treatment since the first prescription); (e) inclusion in the long-term prescriptions program; (f) who had not received any antidepressant treatment within the previous 6 months; and (g) a patient follow-up during a subsequent initiation of treatment.

### Study groups and remission criteria

Patients were divided into two study groups: a) patients with an inadequate response to first-line AD treatment (no remission), and b) patients in remission after the first AD treatment. Patients were followed up for the main outcome measures of the study at 6 and 12 months from the date of start of treatment. Patients were considered to be in remission when they had a Hamilton Depression Rating Scale (HDRS) total score ≤ 7 points after at least 8 weeks of AD treatment in adequate doses [24,25]. Most patients completed the scale. However, the decision to change the intervention strategy was always at the physician´s discretion. The HDRS is routinely performed among patients with depressive symptoms in our centers by GPs or nurses.

The following options were considered as potential strategies for a change of drug treatment: increasing the dose of AD, change to a different AD, combination with a second AD, or association with a new drug without intrinsic antidepressant properties (augmentation) [20].

### Sociodemographic variables and comorbidity

The main study variables were: age (continuous and by ranges) and sex, as well as personal history taken from the International Classification of Primary Care (ICPC-2) [23]. The following were used as summary variable for overall comorbidity for each patient seen: a) the Charlson Comorbidity Index as an approximation to patient severity [26] and b) the Case-mix Index, obtained from the *Adjusted Clinical Groups* (ACG), a system classifying patients by iso-resource consumption [27]. The algorithm of the Grouper ACG® Case-mix System consists of a number of consecutive steps until the 106 mutually exclusive ACG groups are obtained, one for each patient seen. The ACG application provides resource utilization bands (RUBs), so that each patient is grouped into one of the five mutually exclusive categories based on overall morbidity (1: healthy or with a very low morbidity, 2: low morbidity, 3: moderate morbidity, 4: high morbidity, and 5: very high morbidity).

### Drugs prescribed, treatment compliance and persistence, and referrals

Prescriptions of the following therapeutic classes and active ingredients for the central nervous system or psychoactive drugs were considered: ADs (N6A), antipsychotics and mood stabilizers (N5A), anxiolytic drugs (N5B), hypnotics and sedatives (N5C) of the ATC classification [28]. Compliance was defined as the extent of agreement of patient behavior with use of medication based on recommendations by healthcare professionals in charge of the patient. Compliance was estimated as the ratio between the total number of tablets dispensed and the total number of tablets recommended or prescribed, assuming that drug dispensing (purchase of medication at the pharmacy) does not represent actual consumption or intake, but is closely associated with this [29]. Treatment persistence was defined as the time in weeks without discontinuation of initial treatment or without switching to another medication at least 8 weeks after initial prescription. The number of and reasons for referrals to mental health care were analyzed.

### Health and non-health care resources and cost estimation

Direct healthcare costs (direct costs) were those related to care activity (medical visits, diagnostic or therapeutic requests, etc.) performed by professionals, while non-healthcare or indirect costs were those related to work productivity losses. The productivity losses were calculated in terms of days off work [30]. The design of the cost system was defined taking into account the characteristics of the organization and the degree of development of the available information systems. The analytic unit serving as the basis for final calculation (during the study period) was the patient seen, and cost was expressed as mean cost per patient (cost/unit). The different study concepts and their economic assessment are detailed in Table 1 (year 2009). The different rates were obtained from analytical accounting of the centers, except for medication and days of sick leave. Prescriptions (acute, chronic, or demand medical prescriptions) were quantified based on the retail price by pack at the time of prescription. Days of disability for work were considered as non-healthcare costs (indirect costs). The cost was quantified based on the interprofessional minimum wage (source: Spanish Institute of Statistics- INE) [31].

**Table 1:** Details of unit costs of healthcare resources and work productivity losses

**Healthcare and non-healthcare resources**	**Unit cost 2009 (Euros)**
Medical visits in primary care	22.74
Supplemental tests	
Laboratory tests	21.86
Conventional radiology	18.14
Diagnostic/therapeutic tests	36.45
Drug prescription	Retail price/pack
Work productivity-Indirect Costs	
Labor cost per day of sick leave*	79.61

Source of healthcare resources: own analytical accounting. *Source: INE-National Institute of Statistics [31].

### Statistical analysis

A univariate descriptive statistical analysis was performed using the mean, standard deviation, and 95% confidence intervals (CI). Normal data distribution was confirmed using a Kolmogorov-Smirnov test. A nonparametric Kaplan-Meier survival analysis was used to test treatment persistence (median time). ANOVA, Chi-square, and linear Pearson’s correlation tests were used for bivariate analysis. To assess the association of related factors (variables) for each specific strategy, a multinomial logistic regression analysis was performed (procedure: main components). Cost comparison was performed in accordance to recommendations by Thompson and Barber, specifically on the comparison of average health care cost between the study groups [32]. For correction of the cost model was used the analysis of covariance (ANCOVA), with sex, age, comorbidity, and Charlson index as covariates (procedure: estimation of marginal means; Bonferroni correction). SPSSWIN version 17 software was used, and values of p < 0.05 were considered statistically significant.

## Results

**Figure 1:**
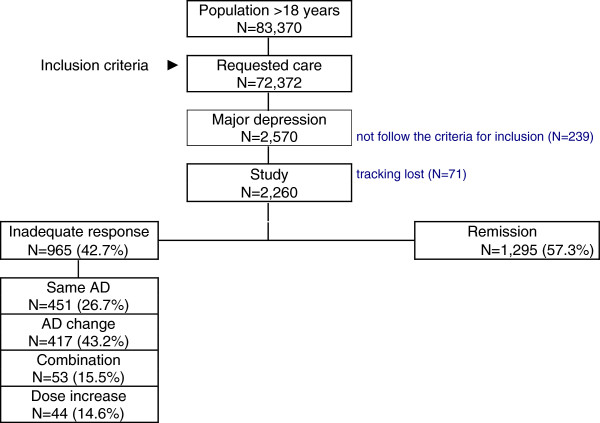
**General study disposition.** Years 2008–2009.

The number of patients >18 years screened was 83,370, of whom 72,372 (86.8%) requested care. Finally, 2,260 subjects who met the inclusion criteria were analyzed (Figure 1). Mean age was 58.8 years (74% females), RUBs were 2.5 points, and mean number of comorbidities was 4.6 per patient. Dyslipidemia (43.6%), high blood pressure (34.2%), and fibromyalgia (25.2%) were the most common comorbidities. Annual incidence (new cases) was 16.3% (95% CI: 14.5-17.5%), with a cumulative incidence rate of 6.8/1,000 inhabitants/year (95% CI: 6.1-7.9/1,000 inhabitants/year).

Forty-three percent of patients (N = 965; 95% CI: 40.0%-46.4%) presented an inadequate response to first-line AD treatment (during patients´ follow-up period: 1 year). Patients without remission were older (61.0 vs. 57.1 years; p < 0.001), females (76.8% versus 71.9%; p = 0,009), and retired (63.1% vs. 47.0%; p < 0.001). These patients had higher mean values of general morbidity (5.3 vs. 4.1 episodes/year) and RUB/year (2.7 versus 2.4) (Table 2).

**Table 2:** Sociodemographic and clinical characteristics

**Groups**	**Inadequate response**	**Remission**	**Total**	**P value**
**Number of patients, %**	**N = 965 (42.7%)**	**N = 1,295 (57.3%)**	**N = 2,260**	
Mean age, years	61.0 (15.1)	57.1 (16.4)	58.8 (15.9)	<0.001
Ranges: 18–44 years	13.7%	24.6%	19.9%	<0.001
45–64 years	46.1%	42.6%	44.1%	<0.001
65–74 years	18.1%	13.8%	15.7%	<0.001
> 74 years	22.1%	19.0%	20.3%	<0.001
Gender, female	76.8%	71.9%	74.0%	0.009
Occupational status, retired	63.1%	47.0%	53.9%	<0.001
Mean number of comorbidities	5.3 (3.5)	4.1 (3.3)	4.6 (3.4)	<0.001
Mean RUBs	2.7 (0.9)	2.4 (1.1)	2.5 (1.1)	<0.001
RUB-1	17.1%	23.4%	20.7%	<0.001
RUB-2	9.3%	18.4%	14.5%	<0.001
RUB-3	59.5%	49.5%	53.8%	<0.001
RUB-4	12.3%	7.1%	9.3%	<0.001
RUB-5	1.8%	1.5%	1.6%	<0,001
Mean Charlson index	0.4 (0.7)	0.3 (0.8)	0.4 (0.7)	NS
*Associated comorbidities*				
Arterial hypertension	39.0%	30.6%	34.2%	<0.001
Diabetes mellitus	17.1%	12.9%	14.7%	0.005
Dyslipidemia	49.7%	39.1%	43.6%	<0.001
Obesity	22.3%	18.7%	20.2%	0.035
Active smoking	22.9%	25.9%	24.6%	NS
Alcoholism	4.2%	4.4%	4.3%	NS
Ischemic heart disease	7.0%	3.7%	5.1%	<0.001
Cerebrovascular events	10.6%	7.7%	8.9%	0.019
Cardiovascular events	15.4%	10.4%	12.6%	<0.001
Organ insufficiency	11.5%	11.1%	11.3%	NS
Bronchial asthma.	8.8%	5.6%	7.0%	<0.001
COPD	4.2%	3.2%	3.6%	NS
Neuropathies	2.4%	1.5%	1.9%	NS
Dementia (all types)	4.9%	3.2%	3.9%	0.032
Organic psychosis	3.5%	2.9%	3.1%	NS
Malignant tumors	10.3%	7.1%	8.5%	0.008
Fibromyalgia	28.9%	22.4%	25.2%	<0.001
Time since MDD onset, years	4.8 (3.8)	3.5 (3.6)	4.1 (3.7)	<0.001
New, incident cases (N = 738)	23.3%	38.7%	32.2%	<0.001
Prevalent cases (N = 1,522)	76.7%	61.3%	67.8%	<0.001

RUBs: resource utilization bands (morbidity burden of patients); COPD: chronic obstructive pulmonary disease; MDD: major depressive disorder; values are given as percentage or mean (standard deviation); NS: not significant.

Patients were considered to be in remission when they had a Hamilton Depression Rating Scale total score ≤ 7 points after at least 8 weeks of AD treatment in adequate doses [24,25].

Summarizing the distribution of different treatment approaches for the management of inadequate AD initial response in naturalistic practice: 43.2% were switched to another AD (time elapsed: 6.5 months), 15.5% were given an additional AD (time elapsed: 5.4 months), AD dose was increased in 14.6% (time elapsed: 6.1 months), and 26.7% remained with the same antidepressant agent (Table 3).

**Table 3:** Pharmacologic strategies

**Groups**	**Same AD**^**1**^	**AD change**^**2**^	**Combination**^**3**^	**Dose increase**^**4**^
**Number of patients, %**	**N = 451 (26.7%)**	**N = 417 (43.2%)**	**N = 53 (15.5%)**	**N = 44 (14.6%)**
*Time to change, months*				
Mean	---	6.5 (3.9)	5.4 (1.9)	6.1 (3.5)
Median	---	5.7	4.0	5.2
Mean age, years	61.9 (15.1)	60.8 (15.0)^‡^	61.1 (15.4)	52.9 (11.4),
Gender, female	75.4%	77.2%	83.1%	79.5%
*Comorbidity*				
Mean number of episodes	5.1 (3.4)	5.5 (3.4)	5.8 (3.9)	5.1 (3.6)
Mean RUBs	2.7 (0.9)	2.7 (0.9)	2.8 (1.0)	2.5 (1.0)
Mean Charlson index	0.4 (0.7)	0.5 (0.7)	0.4 (0.6)	0.3 (0.7)
Duration of MDD, years	4.1 (3.7)	5.2 (3.5)^‡^	6.4 (5.2)	5.1 (3.5)
Incident cases (N = 222)	29.2%,	18.2%	9.8%	20.4%^‡^
Prevalent cases (N = 743)	70.1%	81.5%^‡^	90.1%,	79.5%
Treatment compliance	62.3%,	67.1%	64.8%	69.2%,
Treatment persistence	30.5%,	33.1%	34.8%	43.2%,
*Referrals to psychiatry (N = 199)*				
Referral rate	17.1%^‡^	23.5%	28.2%^‡^	20.5%
Mean referrals per patient	0.2 (0.4)	0.2 (0.5)	0.3 (0.5)^‡^	0.2 (0.5)
Time to referral, months	6.3 (4.1)	6.8 (4.0)	4.4 (3.3)	6.2 (3.8)
*Reasons for referral*				
Disease severity (N = 82)	39.0%	39.8%	53.3%^‡^	55.6%,
No response to treatment (N = 107)	59.7%,	53.1%^‡^	33.3%	44.4%
Patient decision (N = 10)	1.3%	7.1%	13.3%	0.8%
*Cost model (euro)*				
Healthcare costs	782.1	901.4	1,041.8	984.9
Non-healthcare costs (productivity)	1,392.4	1,710.6	1,631.3	1,978.1
Total costs	2,174.4	2,612.0	2,673.1	2,963.1

RUBs: resource utilization bands; values given as percentage or mean (standard deviation); statistical significance: ^‡^p < 0.05 for comparisons between each type of strategy versus the total group with no remission (significance tests: Chi-square and ANOVA; use of post hoc tests). N = 965.

^1^ Same treatment: no changes made in 12 months.

^2^ Antidepressant change.

^3^ Association to another antidepressant.

^4^ Increase in antidepressant dose.

**Figure 2:**
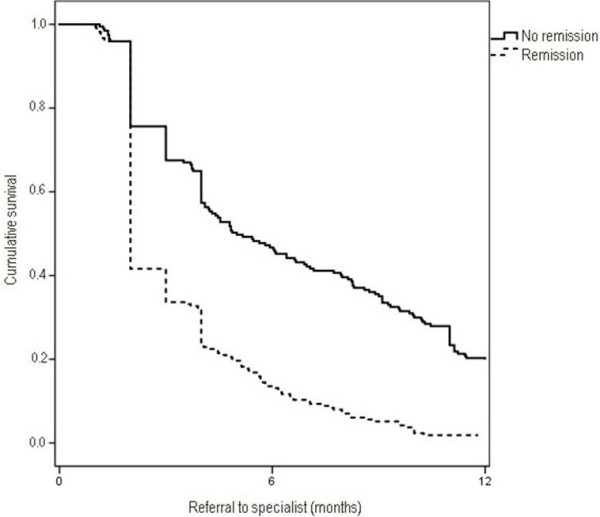
Survival curves of time elapsed to psychiatric referral (B) according to antidepressant response.

Patients with an inadequate AD response had worse percentages of treatment compliance (65.1% versus 67.7%; p < 0.001) and treatment persistence at 12 months (31.8% [95% CI: 27.5%-35.9% versus 53.2% [95% CI: 50.5%-56.9%]; p < 0.001). An acceptable correlation was found between treatment compliance and persistence (r = 0.692; p < 0.001). Referral rate to mental health care was 18.8% (95% CI: 17.2%-20.4%). The reasons were: inadequate response to treatment (47.5%), disease severity (42.1%) and patient´s decision (10.4%). In patients with remission, referral rate was lower (16.8% versus 21.6%; p < 0.001) and time from start of treatment to referral was shorter (3.4 versus 6.4 months; p < 0.001), Figure 2. Table 4 shows the relationship between treatment compliance and persistence, as well as referrals to psychiatry care.

**Table 4:** Relationship between compliance, persistence, and referrals to mental health care. Annual follow-up

**Groups**	**Inadequate response**	**Remission**	**Total**	**P value**
**Number of patients, %**	**N = 965 (42.7%)**	**N = 1,295 (57.3%)**	**N = 2,260**	
*Treatment compliance*				
At 6 months	66.2%	67.9%	67.1%	0.033
At 12 months	65.1%	67.7%	66.6%	<0.001
*Treatment persistence*				
At 6 months	46.5%	64.4%	53.9%	<0.001
At 12 months	31.8%	53.2%	42.7%	<0.001
*Referrals to psychiatry*				
Mean referrals (per patient)	0.2 (0.4)	0.1 (0.4)	0.2 (0.4)	0.005
At 6 months (N = 293)	4.8%	8.2%	13.1%	<0.001
At 12 months (N = 413)	21.6%	16.8%	18.8%	<0.001
Time to referral, months	6.4 (4.0)	3.4 (2.4)	4.8 (3.6)	<0.001
*Reasons for referral*				
Disease severity (N = 174)	41.2%	43.0%	42.1%	<0.001
No response to treatment (N = 196)	53.8%	41.6%	47.5%	<0.001
Patient decision (N = 43)	5.0%	15.4%	10.4%	<0.001

Values given as mean (standard deviation). Treatment compliance: ratio between the numbers of tablets dispensed/prescribed. Persistence: median time without discontinuation of initial treatment or without switch to another medication, at least 8 weeks after initial prescription.

Table 5 shows resource utilization and cost estimation (mean/unit/year) according to AD response. Patients with an inadequate AD response presented higher mean number of visits/year (16.8 vs 11.1; p < 0.001) and days of work disability (20.2 versus 12.8 days; p < 0.001) compared with patients achieving remission. Total annual gross cost of patients demanding care during the 12 months of follow-up amounted to 4.3 million Euros. Healthcare (direct) costs and costs derived from work productivity losses (non-healthcare, indirect costs) represented 32.7% and 67.3% of total costs, respectively (p < 0.001). Medical visits accounted for 16.3%, drugs prescribed for 15.6%, and diagnostic tests for 0.8%. Mean cost per patient of gross direct costs by remission status (absence/presence) was 857.2 versus 443.2 Euros, p < 0,001. In the adjusted model, total costs were 2,668.1 Euros (95%CI: 2,346.9-2,989.2) versus 1,442.6 Euros (95%CI: 1,180.9-1,704.2), respectively; p < 0.001. Costs from work productivity losses were 1,842.0 versus 991.4 Euros, and healthcare (direct) costs were 826.1 versus 451.2 Euros, respectively; p < 0.001. These differences persisted in all cost components (gross and adjusted).

**Table 5:** Use of resources and total cost

**Groups**	**Inadequate response**	**Remission**	**Total**	**P value**
**Number of patients, %**	**N = 965 (42.7%)**	**N = 1,295 (57.3%)**	**N = 2,260 (100%)**	
*Use of resources*				
Medical visits	16.8 (9.1)	11.1 (8.2)	13.6 (8.9)	<0.001
Laboratory tests:	0.6 (0.8)	0.5 (0.7)	0.5 (0.8)	<0.001
Supplemental tests	0.1 (0.4)	0.1 (0.3)	0.1 (0.3)	NS
Referrals	0.2 (0.4)	0.1 (0.4)	0.2 (0.4)	0.005
Work productivity losses (days)	20.2 (63.7)	12.8 (45.5)	16.1 (54.2)	0.001
*Uncorrected cost model (Euros)*				
- Healthcare costs	857.2	443.2	620.0	<0.001
Medical visits	383.9	253.7	309.3	<0.001
Laboratory tests:	15.0	10.3	12.3	<0.001
Supplemental tests	4.3	2.2	3.1	<0.001
Drugs	453.9	177.1	295.3	<0.001
- Non-healthcare costs (productivity)	1,615.3	1,021.5	1,275.1	0.001
Total cost	2,472.5	1,464.8	1,895.0	<0.001
Corrected cost model (Euros)*			*Difference*	
Healthcare costs	826.1	451.2	374.85	<0.001
95% CI	798.5 - 853.5	428.8 - 473.6		
Non-healthcare costs (productivity)	1,842.0	991.4	850.64	<0.001
95% CI	1,525.7 - 2,158.3	733.6 - 1249.1		
Total cost	2,668.1	1,442.6	1,225.49	<0.001
95% CI	2,346.9 - 2,989.2	1,180.9 - 1,704.2		

Values are given as mean (standard deviation); p: statistical significance; CI: confidence interval; Referrals were not considered in the calculation of health costs.

(*) ANCOVA model: each F test contrasts the simple effect of the presence of remission on each combination of levels of the other effects shown. These tests are based on pairwise, linearly independent comparisons between the estimated marginal means. Random components: remission status and sex; covariates: comorbidity and age.

In general, patients undergoing no change in AD were incident cases (29.2%), with low treatment compliance (62.3%) and referral rate (17.1%). Patients under a change to a new AD were prevalent cases (81.5%) with longer disease duration (5.2 years) and referred to psychiatry care due to a lack of response to treatment (53.1%). Patients receiving AD combination had a similar profile. Most of them (90.1%) were prevalent cases with long disease duration (6.4 years). However, they showed a greater referral rate (28.2%), mainly due to disease severity (53.3%), and also have a higher mean healthcare cost (1,041.8 Euros). Patients in whom AD dose was increased were younger (52.9 years) and new cases (20.4%), with high treatment compliance (69.2%).

In the logistic regression model, predictors associated with lack of remission included: treatment non-compliance (OR = 1.7; 95%CI: 1.3-2.7), referral to a psychiatrist (OR = 1.5; 95%CI: 1.3-1.8), years from disease onset (OR = 1.2; 95%CI: 1.1-1.3), age (OR = 1.1; 95%CI: 1.0-1.3), and presence of comorbidity (OR = 1.1; 95%CI: 1.0-1.2); p < 0.05. No variable was associated with a greater probability to the type of strategy used.

## Discussion

In our study, 42.7% of patients presented an inadequate response to first-line antidepressant treatment. AD switch was the most common therapeutic approach (43.2%) performed by GPs after the lack of remission in daily clinical practice. Time until the change of strategy was extremely long (mean 6.5 months) and 26.7% of patients remained with the same initial AD.

Previous studies focused on therapeutic adherence among patients with MDD showed that compliance was low, ranging from 25% to 50% [33-35]. In our study, patients achieving remission showed better rates of compliance (67.7% vs. 65.1%) and treatment persistence (53.2% vs. 31.8%), respectively. Our results are clearly higher, possibly because of the indirect measurement method used [36]. Establishing and maintaining a supporting therapeutic relationship is crucial for ensuring compliance and symptom remission. Factors associated to non-adherence to AD treatment could include lack of information, misguided ideas about mental diseases, lack of family support, cognitive impairment, adverse reactions and side effects, and/or deficient physician-patient communication. Treatment should undoubtedly include, in addition to pharmacologic treatment, individualized interventions with educational and behavioral components [35].

The referral rate to mental health care was 18.8%, mainly due to inadequate response to treatment and disease severity. Kendrick et al. reported an overall 22.8% rate of referrals to a psychologist or psychiatrist, as compared to the 25% and 38% rates reported by Wang and Grembowski, respectively [37-39]. These differences are probably the result of the different factors involved (training of professionals, psychiatric comorbidities, organizational models, resources availability) [40,41].

The most commonly used active ingredients were paroxetine, fluoxetine, sertraline, and citalopram, all of them belonging to the selective serotonin reuptake inhibitors class. These are the antidepressants recommended as first-line treatment in current international guidelines [8,10,14,15].

The different morbidity burdens found in the two groups may have an impact on total costs of the disease. Beyond methodological differences, the results were similar to those of other reviewed studies, although other European investigators have also confirmed their impact by measuring quality of life in these patients [3,5,42,43]. In our study, direct and indirect costs represented 32.7% and 67.3% of total costs, respectively. This distribution is similar to that reported in PC populations in Sweden and Spain, where indirect costs reached 65% of total costs [5,44].

Data related to the different treatment approaches used after an inadequate response to first-line antidepressant treatment, especially in a primary care setting, is one of the strengths of this study. One aspect of these results related to the attitude of GPs which disagree with recommendations in clinical practice guidelines should be stressed: a significant number of patients continued on the same treatment despite not achieving remission. A recent publication by Chang et al. (2012) also found little active management among depressed patients treated in primary care centers in USA [45]. GPs were not more likely to adjust therapy, even when feedback regarding their patients´ symptoms indicated an inadequate response. The STAR-D study showed that patients with longer depressive episodes were less likely to achieve remission [46]. After two treatment steps, it appears that over 50% of patients will achieve remission if they stay in treatment (i.e.,36.8% step 1 plus 30.6% of the remaining 63.2% of patients). Thereafter, the chances of subsequent remission are much lower [46]. Guidelines of scientific associations for the treatment of patients with MDD recommend that, when remission is not achieved with an AD after 6 to 8 weeks of treatment at adequate dose, this should be changed [10]. However, controversy remains and there are no conclusive data as to which is the best alternative available [19,47,48].

The article has several limitations inherent to studies based on population databases, such as disease underreporting or potential variability of professionals in routine use of the different clinical screening scales [49,50]. In addition, the most severe cases were possibly not included in the study because they are usually seen at mental health centers. Potential bias may have resulted from the fact that no consideration was given to the presence or absence of psychotherapeutic interventions in the course of disease. The only direct costs considered were those relating to the public health system and the area of influence of the patient. Sick leaves may in turn be a limited indicator of indirect costs because it underestimates self-employment, and does not take unpaid work into account.

## Conclusions

Our findings demonstrate that the therapeutic strategy most commonly used by GPs for the management of patients with MDD and an inadequate response to first-line treatment is switching to a different antidepressant drug. In addition, there was a significant delay in change of strategy.

Nowadays, symptomatic remission is the main goal in the management of major depressive disorder. Therefore, clinicians should carefully reevaluate patients presenting partial response to AD treatment and to adopt a faster change of pharmacological strategy.

## Competing interests

This study was sponsored by AstraZeneca Spain. ASM and MBT are employees of BSA, the health provider which owned the database which was the subject of this study. JM and LC are employees of AstraZeneca. RNA has no conflicts of interests to declare.

## Authors’ contributions

This was a collaborative work and the authors worked closely each other. All authors participated in the design of the original study or in the interpretation and analysis of data and all of them drafting and have approved the final version of the manuscript. All authors were responsible for literature review and extraction of references, and also for taking the decision to submit the paper for publication.
